# Case study on challenges in research with public partners: A personal data incident during recruitment for a survey study on ageing and housing

**DOI:** 10.1186/s13104-025-07246-8

**Published:** 2025-04-15

**Authors:** Magnus Zingmark, Susanne Iwarsson

**Affiliations:** 1 Department of Health Sciences, Lund University, BMC House E, Box 117, Lund, SE-221 00 Sweden; 2 Department of Community Medicine and Rehabilitation, Umeå University, Umeå, Sweden; 3 Health and Social Care Administration, Municipality of Östersund, Östersund, Sweden

**Keywords:** Transdisciplinary research, User involvement, Co-design, Participatory design, European general data protection regulation (GDPR)

## Abstract

**Objective:**

To highlight experiences from a personal data incident, which occurred during recruitment for a project focused on how housing choices and relocation are related to active and healthy ageing.

**Results:**

Based on established collaboration, the researchers and representatives of housing companies planned for recruitment. Invitations to participate was distributed to persons registered with an interest in relocation. The invitation letter included information according to ethical requirements and a link to an online survey. Within a few days, the housing company was contacted by a person who had received the invitation stating that the company had not secured individual consent to the disclosure of personal data to the researchers. The company and the researchers initiated a range of immediate actions to manage the situation, including a plan for how to respond to persons who wanted their person data to be deleted, how to handle already collected data, and for the continued implementation of the recruitment process. We acknowledge that despite established collaboration based on long term commitment from all parties involved, ethical issues require constant attention. Whereas our case represents a hard-learned lesson on a sensitive ethical issue, the well-established collaboration was of paramount importance for how the situation was handled.

## Introduction

Overall in research on ageing, there is a need for bottom-up approaches involving users [1, 2], in collaboration between researchers and public partners [3]. While transdisciplinary contributions involve positive mechanisms such as joint problem definition, mutual learning, and the sharing of data and information [4], less attention is paid to challenges that might occur. In this paper, the ongoing mixed-methods project Prospective RELOC-AGE [5] serves as the case and provides an example of public involvement in research. Our collaboration involves industry partners, from the proposal stage to communication and implementation. The overall objectives of the project are to study housing choices and relocation and explore effects on active and healthy ageing among people aged 55 + in Sweden being listed with an interest in relocation. To recruit an information rich sample with a sufficient incidence of moves to identify predictors for relocation and associations between aspects of housing, social factors, and active and healthy ageing, we established collaboration with a local public housing company and a national condominium provider. This case study aims to highlight experiences from a personal data incident, which occurred in recruitment for a survey study involving the condominium provider as a partner in research.

### Main text

In an interactive collaborative process, the research team and representatives of the housing companies planned for the recruitment to the Prospective RELOC-AGE Survey, including the establishment of a communication plan. Consulting with lawyers for advice to comply with the European General Data Protection Regulation (GDPR), both companies were advised to set up formal agreements about transfer of personal data between each of them and Lund University (LU). In parallel, the Principal Investigator (PI; SI) secured ethical approval from the Swedish Ethical Review Authority (No. 2020–03457), and the planned personal data register was reported to the data controller at the university according to routine.

Once all formalities were established the partnering condominium provider involved in the project shared their lists of people listed with priority to new establishments (*N* = 23,000) with the research team. The list, including information on personal identity numbers, names, postal and e-mail addresses, and telephone numbers, was stored in a secure system for data storing at the Faculty of Medicine, LU. In March 2021, the research team distributed an invitation letter to participate in the survey to 5,300 of the registered persons, which comprised information about the research project adhering to ethical as well as GDPR principles. In the invitation letter, a link to an online survey was provided where those who agreed to participate provided their informed consent.

### The personal data incident and actions taken

After a few days, the condominium provider’s legal department was contacted by a person who had received the invitation to participate. This was a formal complaint based on the GDPR, stating that the company had not secured individual consent to the disclosure of personal data to LU for research purposes. The company and LU initiated a range of immediate actions to manage the situation. Following legal requirements, the company reported the GDPR incident to the Swedish Authority for Privacy Protection (IMY). In addition, they immediately contacted all persons whose personal data had been disclosed to LU by e-mail, to inform them of the incident and that those who wished to withdraw their personal data should turn to the researchers to have their personal data deleted. As a result, the researchers received e-mails at a very high rate. All those who contacted them requested to be deleted from the recruitment register, and quite a few were upset and angry over the mistake. While most e-mails were received during the first weeks, the flow of e-mails (approx. 600 in all) continued over a period of four months. To be able to handle the situation in practical terms, the PI commissioned a project administrator and a doctoral student to manage the extra and unforeseen workload related to communication and logistics. This included ensuring that data that had been delivered from the condominium provider to LU was deleted in a controlled manner while safeguarding that contact information to those who had already given their informed consent was saved.

The PI (SI) and the project leader (MZ) consulted intensively with lawyers, the data protection officer, the ethical senior consultant as well as with the communication department at LU and informed the faculty Dean and Vice-chancellor about the GDPR incident and possible risks. Early on it was established that LU and the research team had complied with existing laws and regulations; the formal mistake had taken place within the company. Against this background LU established a precise communication strategy to safeguard correct information to media if such attention would escalate. A carefully worded response letter was sent to those who contacted the researchers to have their personal data deleted. At the same time, an e-mail to confirm the deletion of data was prepared, for distribution to each affected individual later.

The partnering company was very keen to correct the mistake and made every effort to minimize harm to the people registered, the research project and researchers, to LU as well as to the company and their employees. They engaged their lawyers and their data protection consultant, and immediately took the initiative to a series of online meetings with the researchers. Later on, the company offered the PI financial compensation to cover costs incurred by the GDPR incident.

Together, the company representatives and the researchers developed a plan for how to handle already collected data and for the continued implementation of the recruitment process. Consulting again with LU’s senior ethical consultant as well as with other experienced researchers, a close examination of the incident focusing on research ethics resulted in the conclusion that it was possible to revise the recruitment process within the scope of the existing approval. The revised process to approach potential participants included information distributed by the housing company to persons on the interest lists about the opportunity to sign up as potential participants via an online portal set up by the research team. Subsequently, potential participants who had provided their contact information received an invitation letter to participate in the survey, information about the research project, and a link to the online survey. Accordingly, albeit with some delay, the recruitment process was resumed and continued, and the survey study data collection was completed in Dec 2021.

In June 2021 the IMY closed the case without any action, with no justification of the decision.

### Critical reflections

The actions initiated immediately once the GDPR incident was recognized were implemented in close collaboration between the researchers and the condominium provider. The research context was grounded in a thematic collaboration initiative at LU, with the goal to encourage collaboration between researchers and the public, involving public partners to generate novel research on ageing and housing from a social rights perspective [6]. We consider this formally established collaboration a prerequisite for how the situation was handled. The collaboration had been ongoing for five years, including mutual learning processes, for example, planning for and conducting seminars, including the identification of problems and research questions related to housing, ageing and health. As a result of such transdisciplinary collaboration [4], there was established communication characterized by mutual respect and understanding between the stakeholders involved.

Whereas transdisciplinary collaboration has several benefits there are challenges as well [7]. Conflicting roles may occur, for example, between the researcher traditionally focused on neutral knowledge production and the collaborator focused on changes in practice, even if sharing a joint ambition to learn more about a phenomenon and contribute to change [8]. To critically reflect on our roles as researchers we can ask ourselves whether we could and should have made further efforts beforehand to safeguard the handling of personal data transferred from the housing companies to the university. As each stakeholder has a responsibility for how the personal data they have access to is handled, we as researchers must accept and respect the independent role of the public partner. Despite the mutual actions initiated in advance to safeguard compliance with the GDPR the incident occurred beyond our control. Based on the case presented in this paper we conclude that public involvement in research may involve joint handling of sensitive situations, even risks in terms of breaking the law or threats to trademarks. Regarding the use of the data collected based on the initial recruitment procedure, the ethical soundness of the project could be questioned. However, according to good scientific practice we had formal ethical approval in place prior to initiating the recruitment process and were obliged to use the data collected in accordance with the specifications in the approval. Extensive efforts were made to inform individuals affected by the GDPR incident about the possibility to delete their personal data and withdraw their informed consent. Thus, ethical requirements were thoroughly handled. Therefore, to not use data that participants who provided informed consent had spent their time and energy on for our data collection would have been fundamentally unethical. Ethical issues obviously need to be continuously monitored, and to make constructive use of our hard-learned experiences we made efforts to share them with other researchers and public partners in presentations and joint seminars.

## Conclusions

Research collaboration involving public partners holds several potential benefits including joint identification and definition of research areas, mutual learning, and utilization of research findings. However, such collaboration also holds the risk for challenging situations and how these can be managed. In this case study, we present how a GDPR incident occurred and was handled during recruitment of participants people 55 + being listed with an interest in relocation because one housing company had not secured individual consent to disclose personal data to the university. We acknowledge that despite established collaboration between academic and public partners based on long term commitment from all parties involved, ethical issues require constant attention. Whereas our case represents a hard-learned lesson on a sensitive ethical issue, the well-established collaboration was of paramount importance for how the situation was handled.

### Limitations

This paper is based on documentation by the researchers during an intense period when acting upon a personal data incident. Thus, there are limited possibilities to generalize the knowledge gained from this project.

## Data Availability

No datasets were generated or analysed during the current study.

## Abbreviations

GDPR: European General Data Protection Regulation
LU: Lund University
PI: Principal Investigator
IMY: Swedish Authority for Privacy Protection
